# Generalized Anxiety Disorder and Social Anxiety Disorder, but Not Panic Anxiety Disorder, Are Associated with Higher Sensitivity to Learning from Negative Feedback: Behavioral and Computational Investigation

**DOI:** 10.3389/fnint.2016.00020

**Published:** 2016-06-29

**Authors:** Hussain Y. Khdour, Oday M. Abushalbaq, Ibrahim T. Mughrabi, Aya F. Imam, Mark A. Gluck, Mohammad M. Herzallah, Ahmed A. Moustafa

**Affiliations:** ^1^Palestinian Neuroscience Initiative, Faculty of Medicine, Al-Quds UniversityJerusalem, State of Palestine; ^2^Center for Molecular and Behavioral Neuroscience, Rutgers UniversityNewark, NJ, USA; ^3^Marcs Institute for Brain and Behavior and School of Social Sciences and Psychology, Western Sydney UniversitySydney, NSW, Australia

**Keywords:** generalized anxiety disorder, social anxiety disorder, panic anxiety disorder, striatum, dopamine, learning, positive feedback, negative feedback

## Abstract

Anxiety disorders, including generalized anxiety disorder (GAD), social anxiety disorder (SAD), and panic anxiety disorder (PAD), are a group of common psychiatric conditions. They are characterized by excessive worrying, uneasiness, and fear of future events, such that they affect social and occupational functioning. Anxiety disorders can alter behavior and cognition as well, yet little is known about the particular domains they affect. In this study, we tested the cognitive correlates of medication-free patients with GAD, SAD, and PAD, along with matched healthy participants using a probabilistic category-learning task that allows the dissociation between positive and negative feedback learning. We also fitted all participants' data to a Q-learning model and various actor-critic models that examine learning rate parameters from positive and negative feedback to investigate effects of valence vs. action on performance. SAD and GAD patients were more sensitive to negative feedback than either PAD patients or healthy participants. PAD, SAD, and GAD patients did not differ in positive-feedback learning compared to healthy participants. We found that Q-learning models provide the simplest fit of the data in comparison to other models. However, computational analysis revealed that groups did not differ in terms of learning rate or exploration values. These findings argue that (a) not all anxiety spectrum disorders share similar cognitive correlates, but are rather different in ways that do not link them to the hallmark of anxiety (higher sensitivity to negative feedback); and (b) perception of negative consequences is the core feature of GAD and SAD, but not PAD. Further research is needed to examine the similarities and differences between anxiety spectrum disorders in other cognitive domains and potential implementation of behavioral therapy to remediate cognitive deficits.

## Introduction

Anxiety disorders are a group of common psychiatric conditions. They are characterized by excessive worry, uneasiness, and fear of future events, such that they affect social and occupational functioning. The DSM-V identifies several forms of anxiety within this spectrum based on distinct phenomenological patterns (Rabe-Jablonska and Bienkiewicz, 1994; APA, 2013) including generalized anxiety disorder (GAD), social anxiety disorder (SAD), and panic anxiety disorder (PAD). Nearly one in three individuals will develop an anxiety spectrum disorder during their lifetime (Kessler et al., 2005). The most common form is specific phobias (lifetime prevalence of 12.5%), followed by SAD (12.1%), GAD (5.7%), and PAD (4.7%) (Kessler et al., 2005; Porcelli et al., 2012).

One of the most important features of anxiety disorders is their cognitive component. This component is exhibited in the form of biased information processing of novel social situations as well as specific and general threats. For instance, the exposure of SAD patients to ordinary social settings induces selective retrieval of past negative social memories. This leads to negative interpretation and response to current events in SAD (Grupe and Nitschke, 2013) as well as PAD (Gladsjo et al., 1998; Dupont et al., 2000; Lautenbacher et al., 2002). This excessive worrying about stimuli and threats may exhibit generalized avoidance and safety behaviors (Clark and Wells, 1995). As a consequence of generalized avoidance, repeated exposure to normal life events produces strong feelings of fear that persist and maintain anxiety for years (Salkovskis et al., 1991; Clark and Wells, 1995; Barlow, 2002).

Despite the significant role of cognitive biases in the psychopathology of anxiety disorders (Mathews and MacLeod, 1994; Beck and Clark, 1997; Heinrichs and Hofmann, 2001), the different cognitive domains, aversively motivated reinforcement learning and instrumental avoidance in particular, are under-investigated (Airaksinen et al., 2005). In comparison, impairments in the different cognitive functions have been extensively explored in the less prevalent anxiety disorders such as Obsessive Compulsive Disorder (Greisberg and McKay, 2003; Kuelz et al., 2004; Muller and Roberts, 2005) and Post-Traumatic Stress Disorder (Golier and Yehuda, 2002; Horner and Hamner, 2002). The majority of studies on cognitive functions in PAD showed impairments in verbal episodic memory and executive functioning (Airaksinen et al., 2005), divided attention (Lautenbacher et al., 2002), short-term verbal memory and learning (Asmundson et al., 1994), verbal long-term memory (Lucas et al., 1991), and visual memory (Lucas et al., 1991; Boldrini et al., 2005). In SAD patients, several studies reported deficits in attentive, executive, and visuo-spatial functions (Cohen et al., 1996), in short-term verbal memory (Asmundson et al., 1994), and in avoidance learning (Ly and Roelofs, 2009). In contrast to these findings, other studies found no evidence of most of these impairments in PAD and GAD (Gladsjo et al., 1998; Purcell et al., 1998a,b). Cognitive impairments in GAD are the least addressed in anxiety disorders. The few studies in this area of research have found no correlation between cognitive deficits and GAD (Zalewski et al., 1994; Airaksinen et al., 2005).

Unfortunately, many of the aforementioned cognitive studies reported general findings with contradictory conclusions. Few studies investigated learning from positive and negative feedback in anxiety disorders. Subjects with high anxiety traits showed higher avoidance of negative feedback compared to healthy controls in the context of Pavlovian and instrumental conditioning (Lovibond et al., 2008; Ly and Roelofs, 2009; Vriends et al., 2012; Cremers et al., 2014).

In neurochemical terms, converging evidence has associated dopamine-dependent pathways in the ventral region of the striatum with motivational processing (Haber and Knutson, 2010). In addition, strong evidence suggests that dopamine and serotonin modulate processing of negative feedback (Henkel et al., 2002; Pariante and Lightman, 2008; Moustafa et al., 2013).

Computational models, such as Q-learning, have been used to fit behavioral data (Frank et al., 2007a; Moustafa and Maida, 2007; Rutledge et al., 2009). More recently, actor-critic models (Barto, 1995; Dayan and Balleine, 2002) have provided good fits to behavioral results (Collins et al., 2014). Computational and experimental investigations argue that the ventral and dorsal striatum (particularly the caudate nucleus) play dissociable roles in learning and decision-making. Specifically, many argue that the critic corresponds to the ventral striatum while the actor corresponds to the dorsal striatum (Cardinal et al., 2002; O'Doherty et al., 2004; Guitart-Masip et al., 2014).

In this study, we examined the cognitive effects of the three subtypes of anxiety disorder on learning from positive versus negative feedback. To our knowledge, this might be the first study to address feedback learning in these three disorders in one study using one cognitive task. We tested patients with GAD, SAD, PAD off their medications along with matched healthy controls, using a category-learning task that allows the dissociation between learning from positive and negative feedback (Bódi et al., 2009; Herzallah et al., 2013). In addition, we applied Q-learning and actor-critic models to behavioral data to test the effects of valence vs. action in learning performance. As in our prior models, the actor-critic model in its most extended form included four free parameters, dealing with both learning from positive and negative feedback in both critic and actor. These parameters are related to learning from positive vs. negative prediction error in the critic as well as learning from positive vs. negative prediction error in the actor. We additionally explored the role played by noise and action selection parameters using various other models.

## Methods

### Participants

We recruited 73 eligible participants from the clinics associated with Cairo and Ain Shams Universities. The participant groups were: GAD (*n* = 18), SAD (*n* = 20), PAD (*n* = 17), or Healthy Controls (HCs; *n* = 18). HC were either partners of patients or were recruited from the community. All participants underwent clinical diagnostic DSM-IV-TR interviews and strictured clinical interviews using the Mini International Neuropsychiatric Interview (MINI; Amorim et al., 1998) to confirm the diagnosis and absence of comorbidities prior to cognitive testing. Participants' age ranged from 30 to 60 years. Participants were group matched for age (*M* = 43.10; *SD* = 5.54), gender (41 males and 32 females), years of education (*M* = 11.93; *SD* = 2.99), and disease duration (*M* = 12.87; *SD* = 3.66) as shown in Table 1. Inclusion criteria for HC included absence of any psychiatric, neurological, or other disorders that might affect cognition.

**Table 1:** **Summary of demographic and neuropsychological results**.

	**Age**	**Education**	**Disease duration**	**HAM-A**	**NAART**	**Forward digit**	**Backward digit**
PAD	41.52 (4.93)	12.06 (2.84)	12.35 (3.41)	22.59 (3.98)	33.24 (11.99)	8.00 (2.00)	6.76 (2.25)
SAD	44.95 (4.27)	12.15 (2.87)	13.75 (3.45)	24.50 (4.72)	34.10 (10.59)	8.10 (1.48)	6.60 (1.93)
GAD	42.11 (5.70)	11.61 (3.25)	12.39 (4.13)	24.50 (2.98)	36.28 (5.80)	7.22 (1.80)	6.17 (1.61)
HC	43.50 (6.84)	11.88 (3.22)		7.50 (3.20)	35.50 (6.84)	8.44 (1.72)	6.61 (1.50)

*Age, education, and disease durations are reported in years. PAD is Panic Anxiety Disorder, SAD is Social Anxiety Disorder, GAD is Generalized Anxiety Disorder, and HC is healthy controls*.

Exclusion criteria for all participants included psychotropic drug exposure; major medical, or neurological illness; illicit drug use or alcohol abuse within the past year; lifetime history of alcohol or drug dependence; psychiatric disorders other than the three anxiety disorders; current pregnancy, or breastfeeding. After receiving a complete description of the study, participants provided written informed consent as approved and conformed by both the Ethics committee and the guidelines for protection of human participants.

### Neuropsychological test battery

All participants completed the Arabic version of neuropsychological tests by the clinicians: the North American Adult Reading Test (NAART; Uttl, 2002), the Wechsler Adult Intelligence Scale—Revised (WAIS-R) Digit Span test (Forward and Backward; Schroeder et al., 2012). Further, all participants completed the Hamilton Anxiety Rating Scale (HAM-A; Hamilton, 1959) to rate the severity of a participant's anxiety.

There was no significant difference between groups in NAART scores (Kruskal-Wallis *H* = 0.201, *df* = 3, *p* = 0.977). However, HAM-A differed significantly across groups (Kruskal-Wallis *H* = 43.731, *df* = 3, *p* < 0.001). Tukey's *HSD* on HAM-A results revealed significant differences between the control group and PAD, SAD and Gad (*p* < 0.001), but no significant differences between the three disease groups (PAD, SAD, and GAD; *p* > 0.05). We used the Kruskal-Wallis test to compare the WAIS forward digit, and WAIS backward digit among groups, which showed no significant effect of group (Forward digit: *H* = 5.071, *df* = 3, *p* = 0.167; Backward digit: *H* = 1.602, *df* = 3, *p* = 0.659), respectively.

### Computer-based cognitive task

#### Learning from positive and negative feedback

**Figure 1:**
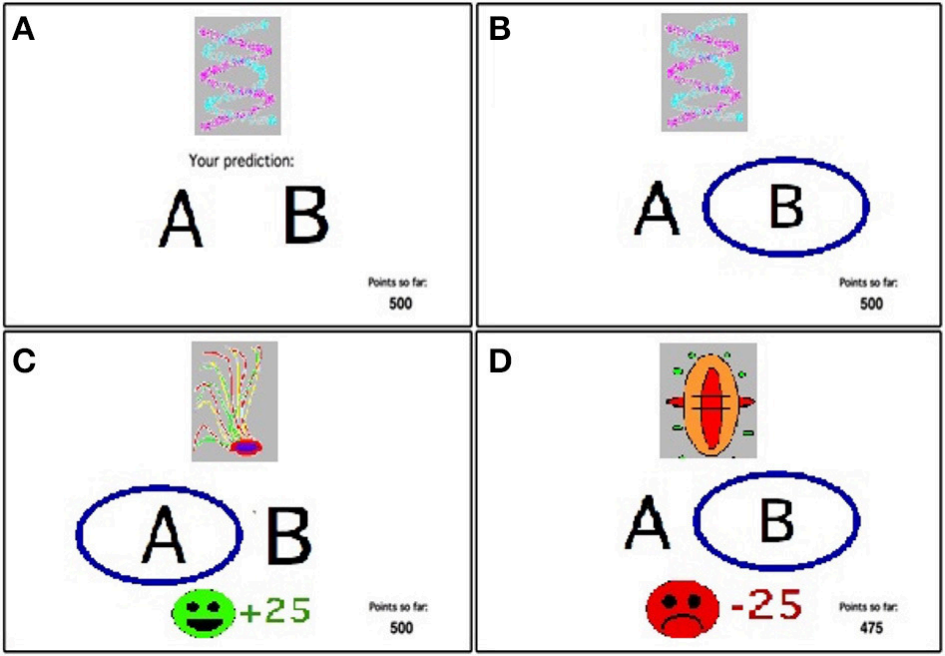
**The feedback-based probabilistic classification task. (A)** On each trial, the participant sees one of four stimuli and is asked whether this stimulus predicts rain or sun. **(B)** No feedback is given for incorrect answers in positive feedback stimuli or correct answers in negative feedback stimuli. **(C)** For positive feedback stimuli, correct responses get rewarded with visual feedback and 25 point winnings. **(D)** For negative feedback stimuli, incorrect responses get punished with visual feedback and the loss of 25 points.

All participants were administered a computer-based probabilistic classification task (Bódi et al., 2009). On each trial, participants viewed one of four images (Figure 1), and were asked to guess whether it belonged to category A or B. On any given trial, stimuli S1 and S3 belonged to category A with 80% probability and to category B with 20% probability, while stimuli S2 and S4 belonged to category B with 80% probability and to category A with 20% probability (Table 2). Stimuli S1 and S2 were used in the positive-feedback learning task. Two stimuli per valence were employed in order to balance category outcome frequencies, so that one stimulus in each task would be associated with each outcome. Thus, if the participant correctly guessed category membership on a trial with either of these stimuli, a positive feedback of +25 points was received; if the participant guessed incorrectly, no feedback appeared. Stimuli S3 and S4 were used in the negative-feedback learning task. Thus, if the participant guessed incorrectly on a trial with either of these stimuli, a negative feedback of −25 was received; correct guesses received no feedback.

**Table 2:** **Category and feedback structure of the probabilistic classification task**.

**Stimulus**	**Probability class A (%)**	**Probability class B (%)**	**Feedback**
S1	80	20	If correct: +25
S2	20	80	If incorrect: no feedback
S3	80	20	If correct: no feedback
S4	20	80	If incorrect: −25

The experiment was conducted on a Macintosh Macbook, programmed in the SuperCard language. The participant was seated in a quiet testing room at a comfortable viewing distance from the screen. The keyboard was masked except for two keys, labeled “A” and “B” that the participant could use to enter responses. Participants first completed a practice phase which walked the participant through an example of a correct and an incorrect response to a sample trial in the negative-feedback learning task and an example of a correct and incorrect response to a sample trial in the positive-feedback learning task. These examples used images other than those assigned to S1–S4. The actual task contained 160 trials, separated and randomized into four blocks. Trials were separated by a 2 s interval, during which time the screen was blank. Within each block, each stimulus appeared 10 times, 8 times with the more common outcome (e.g. category ‘A’ for S1 and S3 and ‘B’ for S2 and S4) and 2 times with the less common outcome. Thus, training on the positive-feedback learning task (S1 and S2) and negative-feedback learning task (S3 and S4) were intermixed. The no-feedback outcome, when it arrived, was ambiguous, as it could signal lack of positive feedback (if received during a trial with S1 or S2) or lack of negative feedback (if received during a trial with S3 or S4). At the end of the 160 trials, if the participant's running tally of points was less than 500 (i.e., no more than the points awarded at the start of the experiment), additional trials were added on which the participant's response was always taken as correct, until the tally was at least 525. This was done in an attempt to minimize frustration in participants by ensuring that all participants terminated the experiment with more points than they had started with. Data from any such additional trials were not analyzed. On each trial, the computer recorded whether the participant made the optimal response (i.e., Category A for S1 and S3 and Category B for S2 and S4) regardless of actual outcome.

### Statistical analysis

The normality of data distribution was checked using Kolmogorov–Smirnov tests; age, disease duration, overall learning from positive feedback, overall learning from negative feedback, and learning from negative feedback in the 4th block were normally distributed (*p* > 0.05) and so ANOVA was conducted. Kruskal-Wallis test was used to analyze the data that were not normally distributed (*p* < 0.05); Education, HAM-A, NAART, Forward digit, Backward digit, and learning from positive feedback in the 4th block.

For the computer-based cognitive task we used mixed-design two way ANOVA, followed by one-way ANOVA and *post-hoc* tests, Bonferroni *post-hoc* tests. The level of significance was set at α = 0.05.

### Computational model

We fit each participant's behavioral data using Q-learning and various actor-critic models (see Table 3 for models used), and used a hierarchical Bayesian procedure for estimating model parameter values for each participant (for more details, see Piray et al., 2014).

**Table 3:** **Computational models used to fit the data from all groups**.

**Model names**	**Description**	**Free parameters**
Actor-critic (free β)	Full actor-critic model	5 ($\alpha_{c}^{+}$, $\alpha_{c}^{-}$, $\alpha_{a}^{+}$, $\alpha_{a}^{-}$, β)
Actor-critic (No β)	Actor-critic model with β = 1	4 ($\alpha_{c}^{+}$, $\alpha_{c}^{-}$, $\alpha_{a}^{+}$, $\alpha_{a}^{-}$)
Actor only	No critic	3 ($\alpha_{a}^{+}$, $\alpha_{a}^{-}$, β)
Q-learning	Q-learning model	2 ($\alpha_{a}^{-}$, β)

The actor-critic models used here had different learning rates for positive and negative prediction errors for updating critic's values and actor's decision. So, the models had two critic's learning rates: $\alpha_{c}^{+}$ and $\alpha_{c}^{-}$; and two actor's learning rates: $\alpha_{a}^{+}$ and $\alpha_{a}^{-}$. The model estimates the value of stimulus-outcome pairs for each participant separately. On each trial *t* the value of the observed stimulus, *s*_*t*_, and the chosen response, *c*_*t*_, is updated according to the following rules:

$$\begin{array}{lll} Q_{t+1}\left(s_{t},a_{t}\right) & = & Q_{t}\left(s_{t},a_{t}\right)+\alpha_{a}^{+}\delta_{t}\text{ if }\delta_{t}>0\\ Q_{t+1}\left(s_{t},c_{t}\right) & = & Q_{t}\left(s_{t},c_{t}\right)+\alpha_{a}^{-}\delta_{t}\text{ if }\delta_{t}>0 \end{array}$$

where α^+^ and α^−^ are the learning rates for positive and negative prediction error, respectively, which determine the degree that recent prediction error affects expected value. Increasing evidence shows that dissociation between learning rates for positive and negative prediction error has a plausible neural substrate (Frank et al., 2007a; Rutledge et al., 2009). δ_*t*_ is the prediction error signal, which is the discrepancy between actual outcome, *o*_*t*_, and expected value:

$$\begin{array}{l} \delta_{t}=o_{t}-Q_{t}\left(c_{t},a_{t}\right) \end{array}$$

The probability of chosen option is then computed using softmax equation:

$$\begin{array}{l} p(c_{t}=A|s_{t})=\frac{1}{\begin{array}{l} 1+\text{exp}[-\beta (Q_{t}(s_{t},A)-Q_{t}(s_{t},B))-\varphi (C_{t}(s_{t},A)\\ \;-\;C_{t}(s_{t},B))] \end{array}}\\ p(c_{t}=B|s_{t})=1-p(c_{t}=A|s_{t}) \end{array}$$

where *p*(*c*_*t*_ = *A*|*s*_*t*_) and *p*(*c*_*t*_ = *B*|*s*_*t*_) are the probabilities of choosing *A* and *B*, respectively. β is a noise parameter. *C*_*t*_(*s*_*t*_, *A*) and *C*_*t*_(*s*_*t*_, *B*) represents the choice of *A* and *B* on the last presentation of *s*_*t*_, respectively. Thus, *C*_*t*_(*s*_*t*_, *A*) = 1 and *C*_*t*_(*s*_*t*_, *B*) = 0 if *A* has been chosen in the previous presentation of *s*_*t*_ before trial *t* and otherwise if *B* has been chosen, *C*_*t*_(*s*_*t*_, *A*) = 0 and *C*_*t*_(*s*_*t*_, *B*) = 1. So, φ determines how much previous choices, independent of reward history, affect current choice. While positive values of φ represent a tendency to perseverate on previous choices, negative values represent tendency to switch more between options. As shown in Table 3, we ran two different actor-critic models (with fixed and variable noise parameters), as well as additional models for comparison.

Formally, the prediction error signal here is computed according to the following equation:

$$\begin{array}{l} \delta_{t}=o_{t}-V_{t}\left(s_{t}\right) \end{array}$$

where *V*_*t*_(*s*_*t*_) is the critic's expected value for *s*_*t*_. Then, the critic's value is updated using prediction error signal:

$$\begin{array}{l} V_{t\;+\;1}\left(s_{t}\right)=V_{t}\left(s_{t}\right)+\alpha_{c}\delta_{t} \end{array}$$

where α_*c*_ is the critic's learning rate. The prediction error is also conveyed to the actor for updating preferences of the actor for the selected choice:

$$\begin{array}{l} P_{t\;+\;1}\left(s_{t},c_{t}\right)=P_{t}\left(s_{t},c_{t}\right)+\alpha_{a}\delta_{t} \end{array}$$

where α_*a*_ is the actor's learning rate. Here, the probability of each choice is computed according to actor's preferences:

$$\begin{array}{l} p(c_{t}=A|s_{t})=\frac{1}{\begin{array}{l} 1+\text{exp}[-\beta (Q_{t}(s_{t},A)-Q_{t}(s_{t},B))-\varphi (C_{t}(s_{t},A)\\ \;-\;C_{t}(s_{t},B))] \end{array}}\\ p(c_{t}=B|s_{t})=1-p(c_{t}=A|s_{t}) \end{array}$$

Further, we fit the data to two simpler models: first, a Q-learning model (Watkins and Dayan, 1992; Sutton and Barto, 1998; Frank et al., 2007b) where there is only one learning rate. The prediction error is therefore calculated based on the values of the stimulus-selected action pair, as opposed to the value of the stimulus independent of the selected action (as in the actor critic model). This model therefore has two free parameters: $\alpha_{a}^{-}$, β.

Now, we describe the procedure of data fitting. For each subject, we fit the data to each of the models described above. We find best parameter values for each subject. The search for parameter values is conducted using Matlab function fmincon.

After parameter values are determined, for each subject under each model, model fit was assessed by computing negative log likelihood estimates (negLLE) to estimate the a priori probability of the data, given a particular combination of parameter values (see Frank et al., 2007b).

$$\begin{array}{l} negLLE=\sum_{t\;=\;1..160}\text{logPr}\left(r,t\right) \end{array}$$

where *r* is the response made by the subject on trial *t* [i.e., *Pr*(*r, t*)] is the probability that the model makes the same response as the subject on that trial). Estimated parameters for each participant (under each model) were defined as the parameter values of that together resulted in the lowest *negLLE* for that participant's data.

To compare models, we used the Bayesian Information Criterion (BIC; Schwartz, 1978; Moustafa et al., 2015a,b; Myers et al., 2016), which penalizes models with more free parameters:

$$\begin{array}{l} BIC=k^{*}\text{ln}\left(n\right)-2^{*}\text{ln}\left(negLLE\right) \end{array}$$

where *k* is the number of free parameters and *n* is the number of observations (here, *n* = 160); lower values of *BIC* indicate fewer explanatory variables, better fit, or both (Table 4). We then used the random effects Bayesian model selection procedure (Stephan et al., 2009; Penny et al., 2010) which takes into account the possibility that different models may have generated different subjects' data, to generate expected posterior probabilities for each model.

**Table 4:** **Value of negLLE and BIC for all models used to fit the data from all groups**.

**Model names**	**negLLE**	**BIC**
Actor-critic (free β)	74.01	16.76
Actor-critic (No β)	75.34	11.65
Actor only	76.11	6.56
Q-learning	77.22	1.45

## Results

### Behavioral results

We used one-sample *t*-test on the mean accuracy across blocks in both positive- and negative-feedback, with Bonferroni corrected α = 0.0125 to protect the level of significance, to ensure that participants learned significantly better than chance in different groups. In positive-feedback learning, participants in all groups learned significantly better than chance except GAD and SAD [GAD: *t*_(17)_ = 2.62, *p* = 0.018; PAD: *t*_(16)_ = 2.87, *p* = 0.011; SAD: *t*_(19)_ = 2.75, *p* = 0.013; HC: *t*_(17)_ = 3.77, *p* = 0.002]. In negative-feedback learning, all groups learned significantly better than chance [GAD: *t*_(17)_ = 9.48, *p* < 0.001; PAD: *t*_(16)_ = 3.23, *p* = 0.005; SAD: *t*_(19)_ = 13.47, *p* < 0.001; HC: *t*_(17)_ = 6.52, *p* < 0.001].

**Figure 2:**
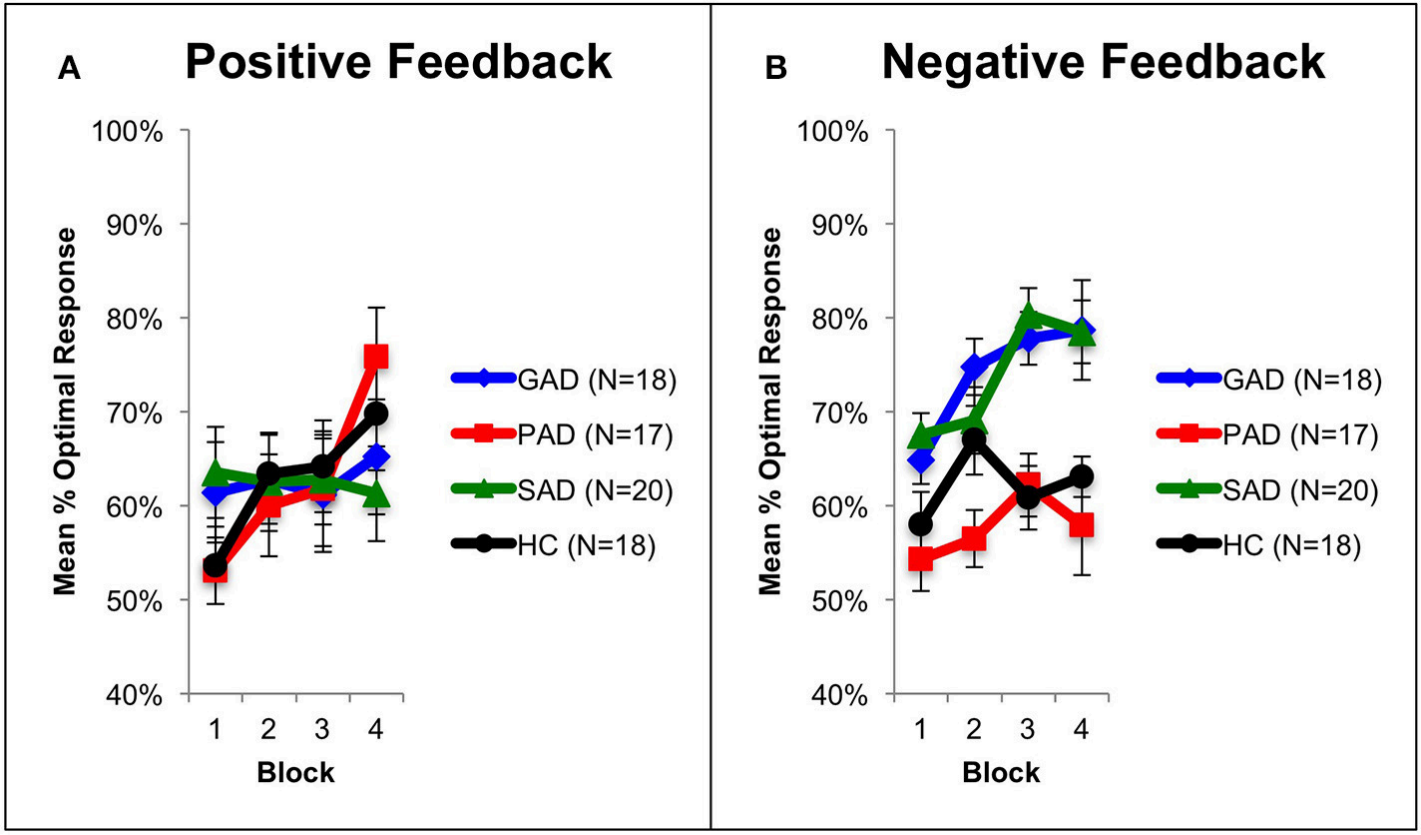
**Performance on the positive and negative feedback learning task. (A)** The mean number of optimal responses in the four phases for positive feedback stimuli (+SEM). **(B)** The mean number of optimal responses in the four phases for negative feedback stimuli (+SEM). PAD is Panic Anxiety Disorder, SAD is Social Anxiety Disorder; GAD is Generalized Anxiety Disorder, and HC is healthy controls.

**Figure 3:**
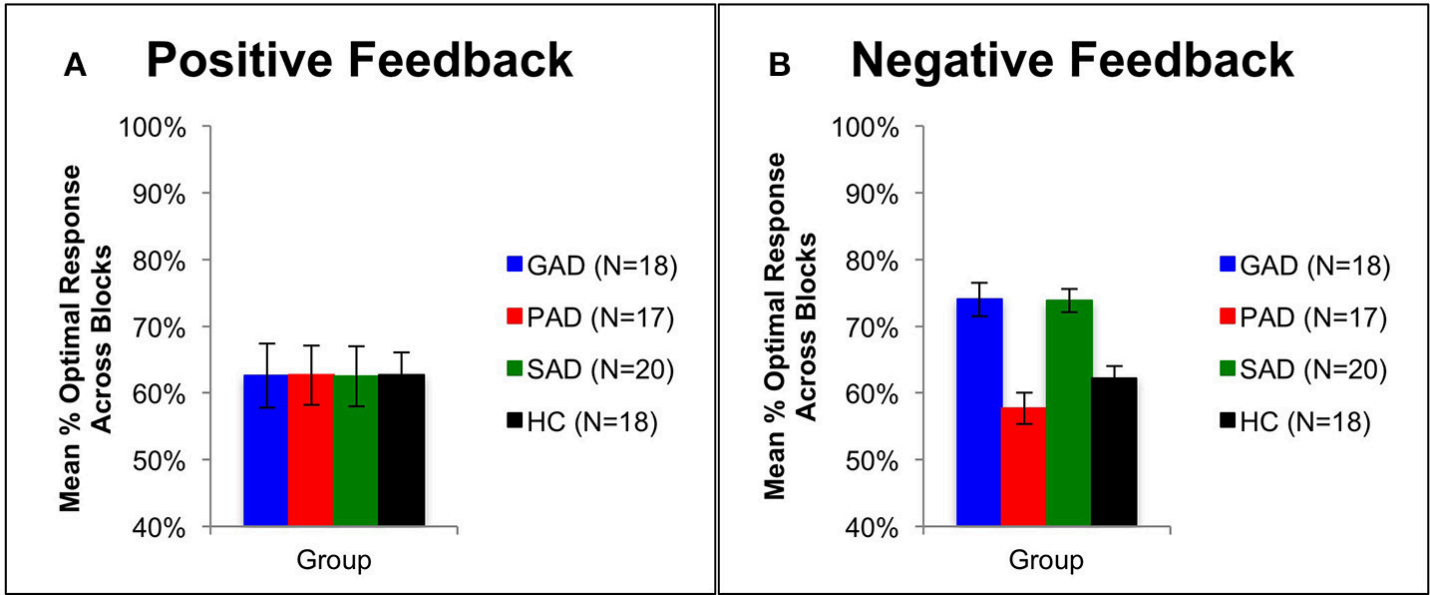
**Performance on the positive and negative feedback learning task. (A)** The mean number of correct responses across blocks for the positive feedback stimuli (+SEM). **(B)** The mean number of correct responses across blocks for the negative feedback stimuli (+SEM). PAD is Panic Anxiety Disorder, SAD is Social Anxiety Disorder; GAD is Generalized Anxiety Disorder, and HC is healthy controls.

Using mixed-design three-way ANOVA, we analyzed the data obtained from the cognitive task with group as the between-subject variable, learning block and feedback type as within-subject variables, and the number of optimal responses on positive and negative-feedback as the dependent variables. There was a significant effect of group [*F*_(3, 69)_ = 3.036, *p* = 0.035, η^2^ = 0.117] and block [*F*_(3, 207)_ = 6.425, *p* < 0.001, η^2^ = 0.085], along with an interaction between feedback type and group [*F*_(3, 69)_ = 2.797, *p* = 0.047, η^2^ = 0.108] and between feedback type, group, and block [*F*_(9, 207)_ = 3.211, *p* = 0.001, η^2^ = 0.122]. However, there was neither a significant effect of feedback type [*F*_(1, 69)_ = 2.683, *p* = 0.106] nor an interaction between block and feedback type [*F*_(3, 207)_ = 1.656, *p* = 0.178] or between group and block [*F*_(9, 207)_ = 1.499, *p* = 0.150]. To examine the interaction between feedback type and group, we used two mixed-design ANOVA *post-hoc* tests to analyze data from positive-feedback and negative-feedback trials separately, with block as the within-subject variable, and group as the between-subject variable. The first mixed-design ANOVA on positive-feedback trials revealed no significant effect of group [*F*_(3, 69)_ = 0.089, *p* = 0.966] and no interaction between group and block [*F*_(3, 207)_ = 1.708, *p* = 0.089], as illustrated in Figures 2A, 3A. However, there was a significant effect of block [*F*_(3, 207)_ = 4.684, *p* = 0.003, η^2^ = 0.064]. Results of the mixed-design ANOVA on negative-feedback trials showed a significant effect of group [*F*_(3, 69)_ = 12.423, *p* < 0.001, η^2^ = 0.351], block [*F*_(3, 207)_ = 3.746, *p* = 0.012, η^2^ = 0.051] and interaction between group and block [*F*_(3, 207)_ = 2.871, *p* = 0.003, η^2^ = 0.111]. Tukey's *HSD post-hoc* analysis revealed a significant difference between GAD and HC, GAD and PAD, SAD and HC, and SAD and PAD (*p* < 0.001), but not between GAD and SAD, or PAD and HC (*p* > 0.5), as illustrated in Figures 2B, 3B.

### Computational results

First, we computed *negLLE* to find model with best fit to the data. Average model fit, defined in terms of *BIC* and *negLLE* is shown for each model in Table 4. Although all models were similarly successful in fitting individual subject data, as indicated by comparable *negLLE*, *BIC* values showed that the Q-learning model provided the simplest fit of the data.

**Figure 4:**
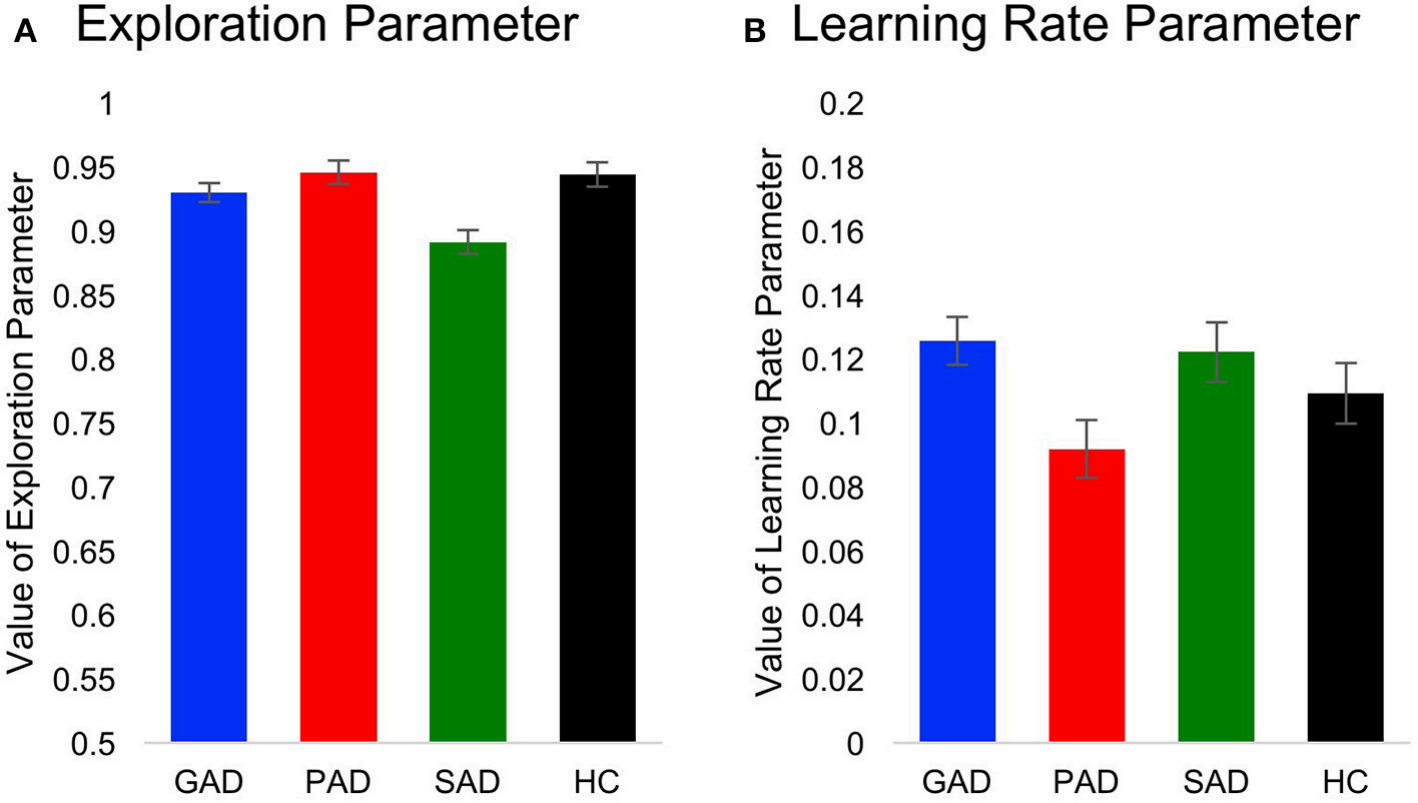
**Computational Q-learning model results**. Here, we show values for **(A)** the learning rate parameter and **(B)** the exploration parameter across groups.

Using two-way mixed-design ANOVA, we analyzed results obtained from fitting of the behavioral data to the Q-learning model. The model parameters (learning rate and exploration) were the within-subject variables, while group (GAD, PAD, SAD, HC) was the between-subject variable. There was a significant difference between the model parameters [*F*_(1, 69)_ = 172.295, *p* < 0.001, η^2^ = 0.714], but neither a significant effect of group [*F*_(3, 69)_ = 0.035, *p* = 0.991] nor significant interaction between model parameters and group [*F*_(3, 69)_ = 0.064, *p* = 0.979]. To further explore the significant difference between parameters, we used two one-way ANOVAs (with Bonferroni corrected α = 0.025 to protect the level of significance) to analyze results from the two model parameters separately with group as the independent variable. There was no effect of group on the learning rate [*F*_(3, 69)_ = 2.201, *p* = 0.096, Figure 4A] or exploration parameter [*F*_(3, 69)_ = 0.036, *p* = 0.991, Figure 4B].

## Discussion

To our knowledge, this is the first study to investigate learning from positive and negative feedback across multiple patient groups with anxiety disorders. We found that SAD and GAD patients learned better from negative feedback than either PAD patients or HC participants. However, patient groups did not differ in positive-feedback learning from HC participants. These results suggest a cognitive dissociation between subtypes of anxiety spectrum disorders, which might underlie a difference in the involved neural circuits in these disorders. Results of our computational Q-learning modeling indicated that enhanced learning from negative feedback in patients with GAD and SAD is not attributed to group differences in speed of learning or ability to explore available outcomes.

### Behavioral and neural correlates of anxiety disorders

We observed differences in participants' performance between block 1 and 4 across the different groups (Figure 2). These differences are evident of an overall learning trend by all participants. In addition, we observed that SAD and GAD patients performed significantly better in negative feedback learning compared to HC. While PAD patients did not differ to HC. Therefore, SAD and GAD patients tend to avoid punishment when exposed to ambiguous stimuli, more than PAD patients do. This behavioral bias (hypersensitivity) toward negative feedback in SAD and GAD patients can be explained by the over-evaluation of the ambiguous stimuli, observed in block 1. This in turn necessitates patients to adopt a negative-feedback avoidance strategy to reach the optimal answer. Eventually, this over-evaluation leads to faster learning by SAD and GAD patients as observed in block 4 (Figure 2B). This implies that SAD and GAD patients utilized avoidance learning as a coping mechanism to reduce exposure to negative feedback and thereby counteract anxiety. These behavioral results are consistent with results reported by Lovibond et al. (2008) indicating that the tendency of healthy participants to utilize avoidance behavior is higher in anxious situations. Along the same lines, Ly and Roelofs (2009) revealed that subjects with high socially anxious displayed increased sensitivity to negative feedback. Further, Cremers et al. (2014) found that SAD patients exhibited increased tendency for anticipation of social punishment than social reward. Additionally, Cremers et al. (2014) also reported higher striatal activation to social punishment when compared to social reward.

Past studies suggested the involvement of VTA and its projections to NAc in the processing of aversive stimuli (Wise, 2004; Schultz, 2006). Further, it was shown that stressful and anxious events activate the mesolimbic dopamine pathway (Tidey and Miczek, 1996; Anstrom and Woodward, 2005; Berton et al., 2006; Brischoux et al., 2009; Ungless et al., 2010). Aversive and stressful events are also clearly capable of enhancing dopaminergic function within the mesolimbic system (Anstrom and Woodward, 2005; Berton et al., 2006; Brischoux et al., 2009; Tidey and Miczek, 1996; Ungless et al., 2010).

Aside from dopamine, Deakin and Graeff's theory proposed that serotonergic neuronal activity in the dorsal raphe nucleus facilitates anxiety-like behavior (Deakin and Graeff, 1991). These modulatory actions of serotonin would only be expressed under stressful situations. Deakin and Graeff's theory conjugates inhibitory avoidance and risk assessment to the symptomatology of GAD and SAD (Deakin and Graeff, 1991). Pharmacological studies have shown that anxiolytic drugs like benzodiazepines are effective in the treatment of GAD (Baldwin and Polkinghorn, 2005). Genetic studies indicate that GAD and SAD patients who carry the low activity allele of the serotonin transporter gene (and hence have higher serotonin) show increased sensitivity to fearful and stressful experiences (Furmark et al., 2004; van der Wee et al., 2008). This is inline with our results showing that GAD and SAD patients learned efficiently from negative feedback.

In our study, PAD patients learned similar to healthy controls from negative feedback, but lower than SAD and GAD patients. Results from PAD patients opposed our conjecture based on our retrospective review of literature. In support of our initial hypothesis, Vriends et al. (2012) showed that patients with phobias exhibited higher susceptibility for avoidance behavior, and more biased discrimination of negative stimuli compared to positive ones. In addition, Asmundson et al. (1994) and Lucas et al. (1991) reported impairments in verbal learning, supported by divided attention deficits in PAD patients (Lautenbacher et al., 2002). However, other studies found no evidence of these impairments in PAD (Gladsjo et al., 1998; Purcell et al., 1998a,b). In the light of previous studies, Deakin and Graeff's theory postulated that serotonergic neuronal activity in the dorsal raphe nucleus facilitates anxiety-like behavior, but inhibits fear (Deakin and Graeff, 1991). Hence, selective serotonin reuptake inhibitors are very effective in the treatment of PAD (Bakker et al., 2005). Genetic studies indicate that PAD patients showed no difference between the two alleles of the serotonin transporter gene in fearful and stressful experiences (Blaya et al., 2007; Strug et al., 2010). The difference in PAD patients performance might be attributed to a heightened fear learning effect that might involve the amygdala and the hippocampus (Gorman et al., 2000), rather than the striatum and VTA (LeDoux et al., 1988, 1990; Davis, 1992; Phillips and LeDoux, 1992).

The three patient groups showed no difference compared to healthy controls in learning from positive feedback (Figure 2A). These results also opposed our predictions, where we expected that anxiety disorder patients would be impaired in positive feedback learning as supported by the literature. Richey et al. (2014) investigated social and non-social reward in SAD patients using fMRI. They found that SAD patients were impaired in both social and non-social rewards. In comparison, NAc was differentially activated in both types of reward, where patients showed NAc hyperactivation in non-social reward and hypoactivation in social reward. Similarly, Cremers et al. (2014) showed that healthy participants exhibited a motivational preference for social reward, which was absent in SAD patients. In addition, ACC connectivity with striatum decreased in reward trials in SAD patients.

Discrepancies in cognitive functions impairments between GAD and SAD patients have been examined in various studies. Neuroimaging and behavioral studies have shown that patients with GAD and SAD had different responses to social-affective stimuli (Becker et al., 2001; Mennin et al., 2005; Blair et al., 2008). GAD patients were slower than SAD patients in recalling emotional words, while SAD patients were impaired on recognition of speech-related words (Becker et al., 2001). Further, patients with GAD usually report greater emotion intensity and fear of experience than patients with SAD, while patients with SAD are less expressive of positive emotions (Mennin et al., 2005). However, our results showed no difference between GAD and SAD patients in learning from positive or negative feedback.

### Computational analysis of cognitive function in anxiety disorders

The benefits of fitting computational models to cognitive data is to disentangle behavioral performance into different components, and thus allows a better understanding of the exact information processing mechanism underlying performance. For example, prior fitting models were applied to neurogenetics (Frank et al., 2007b), neuroimaging (Daw et al., 2006), and animal studies (Beeler et al., 2010).

Fitting our behavioral data using a Q-learning model allowed us to tease apart speed of learning from the ability to explore available options. In other words, abnormal performance in the positive and negative feedback task can be due to either slowness to learn stimulus-outcome associations or stimulus valence, or the inability to explore other available outcomes when the outcome in the current trial is not rewarding. By fitting the Q-learning model to the behavioral data, we found that enhanced learning from negative feedback in patients with GAD and SAD is not attributed to a difference in learning rate or exploration. Our data suggest that enhanced focus on the negative in these anxiety disorders is associated with increased stimulus valence and attentional focus on the negative and not necessarily linked to the speed of learning or ability of explore other available options.

A limitation to our study arises from the fact that all recruited subjects in the SAD, GAD, and PAD groups were tested off medications. With the current dataset, we cannot assess the effect of anxiolytic medications affected on feedback-based learning. Another potential limitation of the current study is the low number of recruited subjects. Future studies, however, should address these limitations and better control for possible confounding variables.

In conclusion, our results argue that not all anxiety spectrum disorders share the same cognitive correlates, but are rather different in ways that do not link them to the hallmark of anxiety (higher sensitivity to negative feedback). Further, research is needed to examine the similarities and differences between anxiety spectrum disorders in other cognitive domains. Further, numerous behavioral studies have shown a similar bias to processing negative feedback in patients with major depressive disorder (Hirschfeld, 2001; Eshel and Roiser, 2010; Herzallah et al., 2013), and major depressive disorder is a common comorbidity with anxiety disorders (Sartorius et al., 1996). Therefore, future studies will examine the effects of comorbid depression on cognitive function in patients with anxiety spectrum disorders.

## Author contributions

HK: Data analysis and manuscript writing. OA: Manuscript writing. IM: Manuscript writing. AI: Data analysis and manuscript writing. MG: Manuscript writing. MH: Data analysis and manuscript writing. AM: Research design, computational modeling, and manuscript writing.

### Conflict of interest statement

The authors declare that the research was conducted in the absence of any commercial or financial relationships that could be construed as a potential conflict of interest. The handling Editor declared a shared affiliation, though no other collaboration, with three of the authors HK, MG, MH, and states that the process nevertheless met the standards of a fair and objective review.
